# Probabilistic Models with Deep Neural Networks

**DOI:** 10.3390/e23010117

**Published:** 2021-01-18

**Authors:** Andrés R. Masegosa, Rafael Cabañas, Helge Langseth, Thomas D. Nielsen, Antonio Salmerón

**Affiliations:** 1 Department of Mathematics, Center for the Development and Transfer of Mathematical Research to Industry (CDTIME), University of Almería, 04120 Almería, Spain; andresmasegosa@ual.es (A.R.M.); antonio.salmeron@ual.es (A.S.); 2 Istituto Dalle Molle di Studi sull’Intelligenza Artificiale (IDSIA), CH-6962 Lugano, Switzerland; 3 Department of Computer Science, Norwegian University of Science and Technology, NO-7491 Trondheim, Norway; helge.langseth@ntnu.no; 4 Department of Computer Science, Aalborg University, DK-9220 Aalborg, Denmark; tdn@cs.aau.dk

**Keywords:** deep probabilistic modeling, variational inference, neural networks, latent variable models, Bayesian learning

## Abstract

Recent advances in statistical inference have significantly expanded the toolbox of probabilistic modeling. Historically, probabilistic modeling has been constrained to very restricted model classes, where exact or approximate probabilistic inference is feasible. However, developments in variational inference, a general form of approximate probabilistic inference that originated in statistical physics, have enabled probabilistic modeling to overcome these limitations: (i) Approximate probabilistic inference is now possible over a broad class of probabilistic models containing a large number of parameters, and (ii) scalable inference methods based on stochastic gradient descent and distributed computing engines allow probabilistic modeling to be applied to massive data sets. One important practical consequence of these advances is the possibility to include deep neural networks within probabilistic models, thereby capturing complex non-linear stochastic relationships between the random variables. These advances, in conjunction with the release of novel probabilistic modeling toolboxes, have greatly expanded the scope of applications of probabilistic models, and allowed the models to take advantage of the recent strides made by the deep learning community. In this paper, we provide an overview of the main concepts, methods, and tools needed to use deep neural networks within a probabilistic modeling framework.

## 1. Introduction

The seminal works about probabilistic graphical models (PGMs) [1,2] made probabilistic modeling an indispensable tool for dealing with uncertainty within many different fields, such as artificial intelligence [3], statistics [4], and machine learning [5,6]. PGMs have been present in the literature for over 30 years and have become a well established and highly influential body of research. At the same time, the problem of computing the posterior probability over hidden quantities given the known evidence, also known as the inference problem [1,2], has been the corner-stone, as well as the bottleneck that defines of the feasibility and applicability of probabilistic modeling (Refs. [7,8] provide in-depth introductions to inference in PGMs).

In the beginning, the first proposed inference algorithms [1,2] were able to compute this posterior in an exact way by exploiting the conditional independence relationships encoded by the graphical structure of the model. However, the set of supported probability distributions was strongly restricted, and mainly multinomial and conditional linear Gaussian distributions were used [2,9]. Researchers quickly realized that the high computational costs of these exact inference schemes made them inappropriate for dealing with the complex stochastic dependency structures that arise in many relevant problems and, consequently, approximate inference methods became a main research focus [8].

Markov Chain Monte Carlo methods were one of the first approximate methods employed for doing inference over complex PGMs [10,11,12]. These techniques are extremely versatile and powerful, and they are able to approximate complex posterior distributions. However, they have serious issues wrt., e.g., the convergence of the underlying Markov chain and poor mixing when approximating high dimensional distributions [10]. Computing such high dimensional posteriors started to become relevant in many domains, specifically when researchers applied a Bayesian approach for learning the parameters of their PGMs from data [5,6,13]. In this setup, the model parameters are treated as unobserved random variables, and the learning problem therefore reduces to computing the posterior probability over the parameters. For models with a large number of parameters, the approach leads to high dimensional posteriors, where the application of Monte Carlo methods becomes infeasible. These issues gave rise to the development of alternative approximate inference schemes.

Belief propagation (BP) [1,14], and the closely related expectation propagation (EP) algorithm [15], have been successfully used to overcome many of the limitations of Monte Carlo methods. These deterministic approximate inference techniques can be implemented using a message-passing scheme that takes advantage of the graph structure of the PGM and, hence, the underlying conditional independence relationships among variables. In terms of distributional assumptions, BP has mainly been used with multinomial and Gaussian distributions. Although EP allows for a more general family of distributions, it is restricted by the need to define a non-trivial quotient operation between the involved densities. While these techniques overcame some of the difficulties of Monte Carlo methods, they presented two new issues: (i) they do not guarantee convergence to an approximate and meaningful solution; and (ii) do not scale to the kind of models that appear in the context of Bayesian learning [6,13]. Again, these challenges motivated researchers to look into alternative approximate inference schemes.

Variational methods [16] were firstly explored in the context of PGMs during the late 90s [17], inspired by their successful application to inference problems encountered in statistical physics. Like BP and EP, they are deterministic approximate inference techniques. The main innovation is to cast the inference problem as a minimization-problem with a well defined loss function, namely the negative evidence lower bound (ELBO) function, which acts as an inference proxy. In general, variational methods guarantee convergence to a local maximum of this ELBO function and therefore to a meaningful solution. By transforming the inference problem into a continuous optimization problem, variational methods can take advantage of recent advances in continuous optimization theory. This was the case for the widely adopted stochastic gradient descent algorithm, which has successfully been used by the machine learning community to scale learning algorithms to big data sets [18]. This same learning algorithm was adapted to variational inference in [19], giving the opportunity to apply probabilistic modeling to problems involving massive data sets. In terms of distributional assumptions, these variational inference methods were restricted to the conjugate exponential family [20], where the gradient of the ELBO wrt. the model parameters can be computed in closed-form [21]. Ad-hoc approaches were developed for models outside this distributional family.

From the start of the field at end of the 1980’s and up to around 2010, probabilistic models had mainly been focused on using distributions from the conjugate exponential family, even though this family of distributions is only able to model linear relationships between the random variables [21]. On the other hand, one of the reasons for the success of deep learning methods (Ref. [22] provides a good introduction to the field) is the ability of deep neural networks to model non-linear relationships among high-dimensional objects, as is, e.g., observed between the pixels in an image or the words in a document. Subsequent advances in variational inference [23,24] enabled the integration of deep neural networks in probabilistic models, thus also making it possible to capture such non-linear relationships among the random variables. This gave rise to a whole new family of probabilistic models, which are often denoted deep generative models [25,26,27,28]. This new family of probabilistic models can encode objects like images, text, audio, and video probabilistically, thus bringing many of the recent advances produced by the deep learning community to the field of probabilistic modeling. The release of modern probabilistic programming languages [29,30,31,32,33] relying on well established deep learning engines like Tensorflow [34] and PyTorch [35] have also significantly contributed to the adoption of these powerful probabilistic modeling techniques.

In this paper, we give a coherent overview of the key concepts and methods needed for integrating deep neural networks in probabilistic models. We do not aim to provide a detailed review of all the methods published in the last years about this topic like in other recent reviews of, e.g., deep generative models [28] and variational inference methods [36]. In contrast, this paper introduces the basic concepts of variational inference methods (Section 2) and neural networks (Section 3). By building on these concepts, in Section 4, we start describing how probabilistic models with deep neural networks are represented in terms of stochastic computational graphs, which is the key data structure for implementing tractable inference methods. In Section 5, we give an overview of the main variational methods used to make inference over this power class of probabilistic models and how they are encoded in terms of a stochastic computational graph. Section 6 provides a brief discussion of current software tools for dealing with probabilistic models containing deep neural networks, all of them take the form a probabilistic programming language [37,38] and are based on the variational inference framework presented here. This paper is also accompanied by online material, where the running examples of the paper together with other basic probabilistic models containing artificial neural networks are implemented to illustrate the presented theoretical concepts and methods (https://github.com/PGM-Lab/ProbModelsDNNs).

## 2. Probabilistic Models within the Conjugate Exponential Family

### 2.1. Latent Variable Models

The conjugate exponential family of distributions [20] covers a broad and widely used range of probability distributions and density functions such as Multinomial, Normal, Gamma, Dirichlet and Beta. They have been used by the machine learning community [5,6,8] due to their convenient properties related to parameter learning and inference tasks.

**Figure 1:**
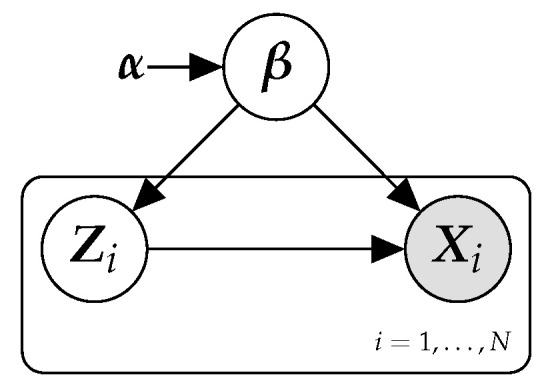
Structure of the probabilistic model examined in this paper, defined for a sample of size *N*.

In the following, we focus on probabilistic graphical models with structure as shown in  Figure 1, and where the full model belongs to the conjugate exponential family. These models are also known as latent variable models (LVMs) [13,39]. LVMs are widely used as a tool for discovering patterns in data sets. The model in  Figure 1 captures “local” patterns, which are specific to sample *i* of the data, using unobservable (or latent) random variables denoted by $Z_{i}$. “Global” patterns, those that are shared among all the samples of the data set, are modeled by means of a set of latent random variables denoted by β. The observed data sample *i*, $X_{i}$, is modeled as random variables whose distribution is conditioned on both the local ($Z_{i}$) and global (β) latent variables. α, a vector of fixed hyper-parameters, is also included in the model.

While the model structure in  Figure 1 at first sight can appear restrictive, it is in fact quite versatile, and many books contain entire sections devoted to LVMs [5,6,8]. For instance, LVMs include popular models like latent Dirichlet allocation (LDA) models used to uncover the hidden topics in a text corpora [40], mixture of Gaussian models to discover hidden clusters in data [5], probabilistic principal component analysis for dimensionality reduction [41], and models to capture drift in a data stream [42,43]. They have been used for knowledge extraction from GPS data [44], genetic data [45], graph data [46], and so on.

The joint distribution of this probabilistic model factorizes into a product of local terms and a global term as

$$p\left(x,z,\beta \right)=p\left(\beta \right)\prod_{i=1}^{N}p\left(x_{i},z_{i}|\beta \right),$$

where *N* is the number of samples. The local latent variables $Z_{i}$ are assumed to be conditionally independent given the global latent variables β.

Another standard assumption in these models is known as the assumption of complete conditional form [19]. Now, the distribution of one latent variable given the other variables in the model can be expressed in exponential family form,

(1) $$\begin{array}{c} \ln p\left(\beta |x,z\right)=h_{g}\left(\beta \right)+\eta_{g}{\left(x,z\right)}^{T}t\left(\beta \right)-a_{g}\left(\eta_{g}\left(x,z\right)\right),\\ \ln p\left(z_{i}|x_{i},\beta \right)=h_{l}\left(z_{i}\right)+\eta_{l}{\left(x_{i},\beta \right)}^{T}t\left(z_{i}\right)-a_{l}\left(\eta_{l}\left(x_{i},\beta \right)\right). \end{array}$$

where the scalar functions $h_{\cdot }\left(\cdot \right)$ and $a_{\cdot }\left(\cdot \right)$ are the base measures and the log-normalizers functions, respectively; the vector functions $\eta_{\cdot }\left(\cdot \right)$ and $t_{\cdot }\left(\cdot \right)$ are the natural parameter and the sufficient statistics vectors, respectively. The subscripts of these functions, here *g* for “global” and *l* for “local”, are used to signify that the different functions differ between variables. The subscripts will be removed when clear from context.

By conjugacy properties, the above assumptions also ensure that the conditional distribution $p(x_{i},z_{i}|\beta )$ is in the exponential family,

(2) $$\ln p\left(x_{i},z_{i}|\beta \right)=\ln h\left(x_{i},z_{i}\right)+\beta^{T}t\left(x_{i},z_{i}\right)-a\left(\beta \right),$$

and, similarly, for the prior distribution p(β),

(3) $$\begin{array}{ccc} \ln p(\beta ) & = & \ln h_{\beta }\left(\beta \right)+\alpha^{T}t_{\beta }\left(\beta \right)-a_{\beta }\left(\alpha \right). \end{array}$$

Combining Equations (2) and (3), we see that the posterior p(β|x,z) remains in the same distribution family as the prior p(β) (that is, we have conjugacy) and, in consequence, the natural parameter of the global posterior $\eta_{g}\left(x,z\right)$ can be expressed as

$$\eta_{g}\left(x,z\right)=\alpha +\sum_{i=1}^{N}t\left(x_{i},z_{i}\right).$$

This representation of the complete conditional will be used later to derive the variational inference scheme over this model.

**Example** **1.** *Principal component analysis (PCA) is a classic statistical technique for dimensionality reduction. It defines a mapping between the d-dimensional data-representation of a point x and its k-dimensional latent representation, z. The latent representation is known as the scores, and the affine transformation is performed using the loading matrix β, which has dimensions k×d.*

*A simplified probabilistic view of PCA [41] is given in Algorithm 1, which provides pseudo-code for the generative process of a probabilistic PCA model. This model is obviously an LVM, as the loadings represented by β are global latent variables and $z_{i}$ is the vector of local latent variables associated with the i-th element in the sample.*

*This model belongs to this conjugate exponential family with complete conditionals, because the joint of p(x,z,β) is multivariate normal and, by standard properties of the multivariate normal distribution, the conditional p(β|z,x) and $p(z_{i}|x_{i},\beta )$ are both conditional multivariate Gaussians. A multivariate normal distribution with mean μ and covariance matrix Σ is a member of the exponential family with natural parameters $\eta ={\left[\Sigma^{-1}\mu ,-1/2\Sigma^{-1}\right]}^{T}$ and sufficient statistics $t\left(x\right)={\left[x,xx^{T}\right]}^{T}$.*

*Note that while this linear relationship between the latent and the observed variables is a strong limitation of this model [47], it guarantees that the model belongs to the conjugate exponential family. Using a non-linear relationship would put PCA outside this model family and would prevent, as we will see in the next section, the use of efficient inference algorithms to calculate p(β|z,x) and $p(z_{i}|x_{i},\beta )$. Similarly, if the variance parameter $\sigma_{x}$ (see Algorithm 1) depends on the latent variables $z_{i}$, the model falls outside the conjugate exponential family.*

**Figure 2:**
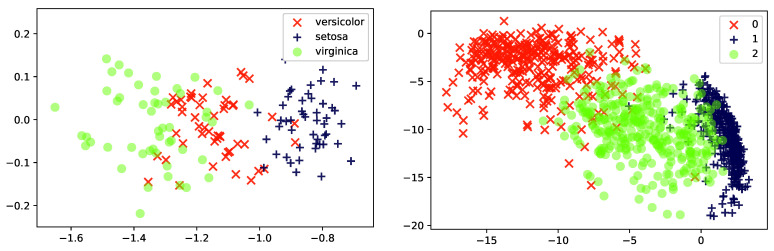
Two-dimensional latent representations resulting of applying a probabilistic PCA of: (**Left**) the iris dataset [48] and (**Right**) a subset of 1000 instances from the MNIST dataset [49] corresponding to the handwritten digits 1, 2 and 3.

*Figure 2 illustrates the behavior of Probabilistic PCA as a feature reduction method on two different data sets, Iris and (a reduced version of) MNIST. The data is projected from data-dimension d=4 (Iris) or d=784 (MNIST) down into k=2 latent dimensions. It can be seen that the method captures some of the underlying structure in the Iris-data, and even generates a representation where the three types of flower can be separated. On the other hand, the MNIST representation appears less informative. Images of the three digits “1”, “2” and “3” are given to the PCA, but even though these three groups of images are quite distinct, the learned representation is not able to clearly separate the classes from one another. As we will see later in this paper, when we consider a more expressive mapping between the local latent $Z_{i}$ and $X_{i}$ (using artificial neural networks), the latent representations will become more informative.*

**Algorithm 1** Pseudo-code of the generative model of a probabilistic PCA model.
# Sample from global random variables$\beta_{u,v}∼N\left(0,1\right)$    # Sample for u=1,…,k, v=1,…,d.
**for** i=1,…,N **do**
 # Sample from the local latent variables $z_{i}∼N\left(0,I\right)$
 # Sample from the observed variables $x_{i}∼N\left(\beta^{T}z_{i},\sigma_{x}^{2}I\right)$

**end for**



### 2.2. Mean-Field Variational Inference

The problem of Bayesian inference reduces to computing the posterior over the unknown quantities (i.e., the global and local latent variables β and z, respectively) given the observations,

$$p\left(\beta ,z|x\right)=\frac{p(x|z,\beta )p(z|\beta )p(\beta )}{\int \int p(x|z,\beta )p(z|\beta )p(\beta )dzd\beta }.$$

Computing the above posterior is intractable for many interesting models, because it requires to solve the complicated multidimensional integral in the denominator. As commented in the introduction, variational inference (VI) methods are one of the best performing options to address this problem. In this section, we revise the main ideas behind this approach.

**Example** **2.** *Computing p(β,z|x) for the probabilistic PCA model described in Example 1 is not feasible since the integral*

p(x)=∫∫p(x|z,β)p(z|β)p(β)dzdβ

*is intractable. The source of the problem is that p(x|β)=∫p(x|z,β)p(z|β)dz is not in the conjugate exponential family.*

Variational inference is a deterministic technique that finds a tractable approximation to an intractable (posterior) distribution. We will use *q* to denote the approximation, and use *p* to signify the true distribution (like p(β,z|x) in the example above). More specifically, let Q denote a set of possible approximations *q*. Now, VI solves the following optimization problem:

(4) $$\min_{q\in Q}KL\left(q||p\right),$$

where KL denotes the Kullback–Leibler divergence between two probability distributions. For the specific problem at hand, this general formulation is more precisely written as

$$\min_{q\left(\beta ,z\right)\in Q}KL\left(q\left(\beta ,z\right)||p\left(\beta ,z|x\right)\right)$$

Notice that while *q* depends on the observations x, it is customary to make this implicit in the notation, and write, e.g., $q\left(\beta ,z\right)$ instead of $q\left(\beta ,z|x\right)$. In practice, one will typically posit that Q is a convenient distributional family indexed by some parameters, say θ, and the minimization of Equation (4) amounts to finding the parameters $\theta^{⋆}$ that minimize the KL divergence.

Under the mean field variational approach, the approximation family Q is assumed to fully factorize. Following the notation in [19], we have that

(5) $$q\left(\beta ,z|\lambda ,\phi \right)=q\left(\beta |\lambda \right)\prod_{i=1}^{N}q\left(z_{i}|\phi_{i}\right),$$

where λ parameterizes the variational distribution of β, while $\phi_{i}$ has the same role for the variational distribution of $Z_{i}$.

Furthermore, if the model is within the conjugate exponential family, each factor in the variational distribution is assumed to belong to the same family of the model’s complete conditionals (see Equation (1)),

(6) $$\begin{array}{c} \ln q\left(\beta |\lambda \right)=h\left(\beta \right)+\lambda^{T}t\left(\beta \right)-a\left(\lambda \right),\\ \ln q\left(z_{i}|\phi_{i}\right)=h\left(z_{i}\right)+\phi_{i}^{T}t\left(z_{i}\right)-a\left(\phi_{i}\right). \end{array}$$

To solve the minimization problem in Equation (4), the variational approach exploits the transformation

(7) lnp(x)=L(λ,ϕ)+KL(q(β,z|λ,ϕ)||p(β,z|x)),

where L can be expressed as

(8) $$L\left(\lambda ,\phi \right)=E_{q}\left[\ln p\left(x,z,\beta \right)\right]-E_{q}\left[\ln q\left(\beta ,z|\lambda ,\phi \right)\right].$$

L is of interest in its own right. Notice in particular that L in Equation (7) is a *lower bound* of lnp(x) since the KL-divergence is non-negative. For this reason, L is usually referred to as the ELBO (evidence lower bound). Furthermore, as lnp(x) is constant in the optimization wrt. *q*, minimizing the KL divergence in Equation (4) is equivalent to maximizing the lower bound L. Variational methods maximize L using gradient based techniques.

The key advantage of having a conjugate exponential model is that the gradients of L wrt. its parameters can always be computed in closed form [21]. This is important, as it leads to a natural scheme in which the parameters are updated iteratively: For a parameter $\theta_{j}$, simply choose the value $\theta_{j}^{⋆}$ so that ${\left.\nabla_{\theta_{j}}L\left(\theta \right)\right|}_{\theta :\theta_{j}=\theta_{j}^{⋆}}=0$. In practice, it is beneficial to use the natural gradient, which is the standard gradient pre-multiplied by the inverse of the Fisher information matrix, to account for the Riemannian geometry of the parameter space [50].

The gradients with respect to the variational parameters λ and ϕ can be computed as follows,

(9) $$\begin{array}{cc} \nabla_{\lambda }^{nat}L\left(\lambda ,\phi \right) & =\alpha +\sum_{i=1}^{N}E_{Z_{i}}\left[t\left(x_{i},Z_{i}\right)\right]-\lambda ,\\ \nabla_{\phi_{i}}^{nat}L\left(\lambda ,\phi \right) & =E_{\beta }\left[\eta_{l}\left(x_{i},\beta \right)\right]-\phi_{i}, \end{array}$$

where $\nabla^{nat}$ denotes natural gradients and $E_{z_{i}}\left[\cdot \right]$ and $E_{\beta }\left[\cdot \right]$ denote expectations with respect to $q(z_{i}|\phi_{i})$ and q(β|λ), respectively.

From the above gradients, we can derive a coordinate ascent algorithm to optimize the ELBO function with the following coordinate ascent rules,

(10) $$\begin{array}{cc} \lambda^{⋆} & =\arg\max_{\lambda }L\left(\lambda ,\phi \right)=\alpha +\sum_{i=1}^{N}E_{Z_{i}}\left[t\left(x_{i},Z_{i}\right)\right],\\ \phi_{i}^{⋆} & =\arg\max_{\phi_{i}}L\left(\lambda ,\phi \right)=E_{\beta }\left[\eta_{l}\left(x_{i},\beta \right)\right]. \end{array}$$

By iteratively running the above updating equations, we are guaranteed to (i) monotonically increase the ELBO function at every time step and (ii) to converge to a stationary point of the ELBO function or, equivalently, the function minimizing Equation (4).

**Example** **3.** *For the PCA model in Example 1, the variational distributions are*

$$\begin{array}{ccc} q(\beta |\mu_{\beta },\Sigma_{\beta }) & = & N(\beta |\mu_{\beta },\Sigma_{\beta }),\\ q(z_{i}|\mu_{z_{i}},\Sigma_{z_{i}}) & = & N(z_{i}|\mu_{z_{i}},\Sigma_{z_{i}}). \end{array}$$

*Given the above variational family, the coordinate updating equations derived from Equation* (10) *can be written, after some algebraic manipulations, as [5]*

$$\begin{array}{ccc} \Sigma_{\beta } & = & {\left(\sum_{i=1}^{N}E\left[Z_{i}Z_{i}^{T}\right]+\sigma_{x}^{2}A\right)}^{-1},\\ \mu_{\beta } & = & {\left[\sum_{i=1}^{N}x_{i}E\left[Z_{i}\right]\right]}^{T}\Sigma_{\beta },\\ \Sigma_{z_{i}} & = & {\left(I+\mu_{\beta }^{T}\mu_{\beta }/\sigma_{x}^{2}\right)}^{-1},\\ \mu_{z_{i}} & = & \Sigma_{z_{i}}\mu_{\beta }^{T}x_{i}/\sigma_{x}^{2}, \end{array}$$

*where A is a diagonal matrix with element at index (i,i) given by $d/\mu_{\beta ,i}^{T}\mu_{\beta ,i}$. Again, we have a set of closed-form equations that guarantees convergence to the solution of the inference problem. We should note that this is possible due to the strong assumptions, imposed both on the probabilistic model p and on the family of variational approximations Q.*

### 2.3. Scalable Variational Inference

Performing VI on large data sets (measured by the number of samples, *N*) raises many challenges. Firstly, the model itself may not fit in memory, and, secondly, the cost of computing the gradient of the ELBO with respect to λ linearly depends on the size of the data set (see Equation (9)), which can be prohibitively expensive when *N* is very large. Stochastic variational inference (SVI) [19] is a popular method for scaling VI to massive data sets, and relies on stochastic optimization techniques [18,51].

We start by re-parameterizing the ELBO so that L is expressed only in terms of the global parameters λ. This is done by defining

(11) $$L\left(\lambda \right)=L(\lambda ,\phi^{⋆}\left(\lambda \right)),$$

where $\phi^{⋆}\left(\lambda \right)$ is defined as in Equation (10), i.e., it returns a local optimum of the local variational parameters ϕ for a given λ. Now L(λ) has the following form:

(12) $$\begin{array}{cc} L(\lambda ) & =E_{q}\left[\ln p\left(\beta \right)\right]-E_{q}\left[\ln q\left(\beta |\lambda \right)\right]\\ \; & +\sum_{i=1}^{N}\max_{\phi_{i}}\left\{E_{q}\left[\ln p\left(x_{i},Z_{i}|\beta \right)\right]-E_{q}\left[\ln q\left(z_{i}|\phi_{i}\right)\right]\right\} \end{array}$$

As shown in [19], we can compute the gradient of L(λ) by first finding $\phi^{⋆}\left(\lambda \right)$, and then compute the gradient w.r.t. λ while keeping $\phi^{⋆}\left(\lambda \right)$ fixed (because $\nabla_{\lambda }L\left(\lambda \right)=\nabla_{\lambda }L\left(\lambda ,\phi^{⋆}\left(\lambda \right)\right)$). By exploiting properties of the conjugate exponential family, Ref. [19] computes the the natural gradient with respect to λ in closed-form as

$$\nabla_{\lambda }^{nat}L\left(\lambda \right)=\alpha +\sum_{i=1}^{N}E_{q(z_{i}|\phi_{i}^{⋆})}\left[t\left(x_{i},Z_{i}\right)\right]-\lambda .$$

The key idea behind SVI is to compute unbiased albeit noisy estimates of $\nabla_{\lambda }^{nat}L$, denoted ${\hat{\nabla }}_{\lambda }^{nat}L$, by randomly selecting a mini-batch of *M* data samples, and then define

$$\begin{array}{c} {\hat{\nabla }}_{\lambda }^{nat}L\left(\lambda \right)=\alpha +\frac{N}{M}\sum_{m=1}^{M}E_{q(z_{i}|\phi_{i}^{⋆})}\left[t\left(x_{i_{m}},Z_{i_{m}}\right)\right]-\lambda , \end{array}$$

where $i_{m}$ is the variable index form the subsampled mini-batch. It is immediate that $E\left[{\hat{\nabla }}_{\lambda }^{nat}L\right]=\nabla_{\lambda }^{nat}L$, hence the estimator is unbiased. Utilizing stochastic optimization theory [51], the ELBO can be optimized by following noisy estimates of the gradient,

(13) $$\begin{array}{c} \lambda_{t+1}=\lambda_{t}+\rho_{t}{\hat{\nabla }}_{\lambda }^{nat}L\left(\lambda_{t}\right), \end{array}$$

where the learning rate $\rho_{t}$ should satisfy the Robbins–Monro conditions (A sequence ${\left\{\rho_{t}\right\}}_{t=1}^{\infty }$ satisfies the Robbins–Monro conditions if $\sum_{t=1}^{\infty }\rho_{t}=\infty$ and $\sum_{t=1}^{\infty }\rho_{t}^{2}<\infty$).

To choose the size of the mini-batch *M*, two conflicting issues should be considered: smaller values of *M* (i.e., M≪N) lead to a reduction in the computational complexity of computing the gradient, while larger values of *M* (i.e., M≫1) reduce the variance of the estimator. The optimal value for *M* is problem dependent [52].

Alternative ways to scale up variational inference in conjugate exponential models involve the use of distributed computing clusters. For example, it can be assumed that the data set is stored in a distributed way among different machines [53]. Then, the problem of computing the ELBO’s gradient given in Equation (9) is scaled up by distributing the computation of the gradient $\nabla_{\phi_{i}}^{nat}L\left(\lambda ,\phi \right)$, so that each machine computes this term for those samples that are locally stored. Finally, all the terms are sent to a master node, which aggregates them and computes the gradient $\nabla_{\lambda }^{nat}L\left(\lambda ,\phi \right)$ (see Equation (9)).

**Example** **4.** *For Example 3, we detailed the variational updating equations for the probabilistic PCA model introduced in Example 1. In order to update $\mu_{\beta }^{⋆}$, we need to iterate over the whole data set. Furthermore, the number of local variational parameters $\mu_{z_{i}}^{⋆}$ and $\Sigma_{z_{i}}^{⋆}$ is equal to the number of data points. Therefore, if N is very large, the computation of these variational updating equations becomes infeasible.*

*Following the methodology presented in this section, we can obtain a new set of variational updating equations,*

$$\begin{array}{ccc} \Sigma_{\beta ,t+1} & = & {\left[\left(1-\rho_{t}\right)\Sigma_{\beta ,t}^{-1}+\rho_{t}\left(\frac{N}{M}\sum_{m=1}^{M}E_{t,i_{m}}\left[Z_{i_{m}}Z_{i_{m}}^{T}\right]+\sigma_{x}^{2}A\right)\right]}^{-1},\\ \mu_{\beta ,t+1} & = & \left(1-\rho_{t}\right)\mu_{\beta ,t+1}+\rho_{t}{\left(\frac{N}{M}\sum_{m=1}^{M}x_{i_{m}}E_{t,i_{m}}\left[Z_{i_{m}}\right]\right)}^{T}\Sigma_{\beta ,t+1}, \end{array}$$

*where $\left\{i_{1},\ldots ,i_{M}\right\}$ are the indexes of the mini-batch, and $E_{t,i_{m}}\left[\cdot \right]$ denotes expectations when $Z_{i_{m}}$ follows a multivariate normal distribution with parameters*

$$\begin{array}{ccc} \Sigma_{t,z_{i_{m}}} & = & {\left(I+\sigma_{x}^{-2}\mu_{\beta ,t}^{T}\mu_{\beta ,t}\right)}^{-1},\\ \mu_{t,z_{i_{m}}} & = & \Sigma_{t,z_{i_{m}}}\mu_{\beta ,t}^{T}x_{i_{m}}/\sigma_{x}^{2}; \end{array}$$

*confer also Example 3. Using this set-up, we do not need to go thorough the full data set to get an update on the global variational parameters.*

### 2.4. Variational Message Passing

So far, we have treated the set of variables x, z and β as undividable blocks of variables without internal structure. However, as we are dealing with flexible probabilistic graphical models, these sets of variables can often encode conditional independencies that can be further exploited when using VI. variational message passing (VMP) [21] is a VI scheme which readily exploits such conditional independencies when performing approximate inference. Now, $Z_{i}$ and $X_{i}$, the set of latent and observable variables associated to the *i*-th data sample, are separated into individual variables $Z_{i}=\left\{Z_{i,1},\ldots ,Z_{i,K}\right\}$, and similarly for $X_{i}$. Additionally, β is regarded as a set of *J* separate random variables $\beta =\{\beta_{1},\ldots ,\beta_{J}\}$. Now, under the mean field assumption, the variational distribution is expressed as

$$q\left(\beta ,z|\lambda_{i},\phi \right)=\prod_{j=1}^{J}q\left(\beta_{j}|\lambda_{j}\right)\prod_{i=1}^{N}\prod_{k=1}^{K}q\left(z_{i,k}|\phi_{i,k}\right).$$

Using the VMP scheme, the gradients wrt. the variational parameters can be computed using a message-passing algorithm which exploits the conditional independencies between the variables in $X_{i}$, $Z_{i}$ and β. The flow of messages is similar to the one employed by loopy belief propagation [1]. The messages are expected sufficient statistics of the variables involved, and since the model is in the conjugate exponential family, both the messages and the update rules can be expressed analytically, leading to parameter updates akin to Equation (10); cf. [21] for details.

## 3. Deep Neural Networks and Computational Graphs

### 3.1. Deep Neural Networks

An artificial neural network (ANN) [54] can be seen as a deterministic non-linear function f(·:W) parametrized by a matrix W. An ANN with *L* hidden layers defines a mapping from a given input x to a given output y. This mapping is built by the recursive application of a sequence of (non-)linear transformations,

(14) $$\begin{array}{ccc} h_{0} & = & r_{0}\left(W_{0}^{T}x\right),\\ & \ldots & \\ h_{l} & = & r_{l}\left(W_{l}^{T}h_{l-1}\right),\\ & \ldots & \\ y & = & r_{L}\left(W_{L}^{T}h_{L-1}\right), \end{array}$$

where $r_{l}\left(\cdot \right)$ defines the (non-linear) activation function at the *l*-th layer; standard activation functions include the *soft-max* function and the *relu* function [55,56]. $W_{l}$ are the parameters defining the linear transformation at the *l*-th layer, where the dimensionality of the target layer is defined by the size of $W_{l}$. Deep neural networks (DNNs) is a renaming of classic ANNs, with the key difference that DNNs usually have a higher number of hidden layers compared to what classical ANNs used to have.

Learning a DNN from a given data set of input-output pairs (x,y) reduces to solving the optimization problem

(15) $$W^{⋆}=\arg\min_{W}\sum_{i=1}^{N}\ell \left(y_{i},f\left(x_{i};W\right)\right),$$

where $\ell (y_{i},{\hat{y}}_{i})$ is a loss function which defines the quality of the model, i.e, how well the output ${\hat{y}}_{i}=f\left(x_{i};W\right)$ returned by the DNN model matches the real output $y_{i}$. This continuous optimization problem is usually solved by applying a variant of the stochastic gradient descent method, which involves the computation of the gradient of the loss function with respect to the parameters of the ANN, $\nabla_{W}\ell \left(y_{i},f\left(x_{i};W\right)\right)$. The algorithm for computing this gradient in an ANN is known as the *back-propagation* algorithm, which is based on the recursive application of the chain-rule of derivatives and typically implemented based in the computational graph of the ANN. A detailed and modern introduction to this field is provided in [22].

### 3.2. Computational Graphs

**Figure 3:**
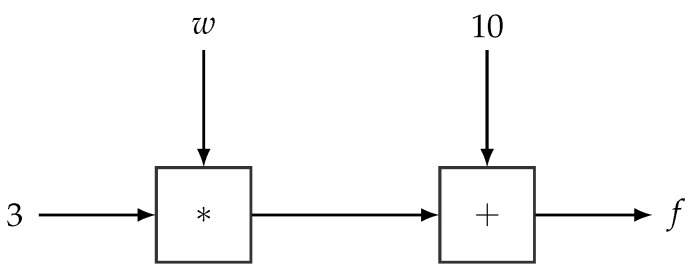
Example of a simple Computational Graph. Squared nodes denote operations, and the rest are input nodes. This computational graph encodes the operation f=3·w+10, where *w* is a variable wrt. which we can differentiate.

Computational graphs [34,35,57] have been extremely useful when developing algorithms and software packages for neural networks and other models in machine learning. The main idea of a computational graph is to express a (deterministic) function, as is the case of a neural network, as an acyclic directed graph defining a sequence of computational operations. A computational graph is composed of input and output nodes as well as operation nodes. The data and the parameters of the model serve as input nodes, whereas the operation nodes (represented as squares in the subsequent diagrams) correspond to the primitive operations of the network and also define the output of the network. The directed edges in the graph specify the inputs of each node. Input nodes are usually defined over tensors (*n*-dimensional arrays) and operations are thus similarly defined over tensors, thereby also enabling the computational graph to, e.g., process batches of data. Figure 3 shows a simple example of a computational graph.

With computational graphs, simple/primitive functions can be combined to form complex operations, and the vast majority of current neural networks can be defined using computational graphs. However, the key strength of computational graphs is that they allow for automatic differentiation [58]. As shown in the previous section (see Equation (15)), most neural network learning algorithms translate to a continuous optimization problem of a differentiable loss function often solved by a gradient descent algorithm. Automatic differentiation is a technique for automatically computing all the partial derivatives of the function encoded by a computational graph: once the graph has been defined using underlying primitive operations, derivatives are automatically calculated based on the “local” derivatives of these operations and the recursive application of the chain rule of derivatives, incurring only a small computational overhead. Before the use of computational graphs in deep learning, these derivatives had to be computed manually, giving rise to a slow and error-prone development process.

**Figure 4:**
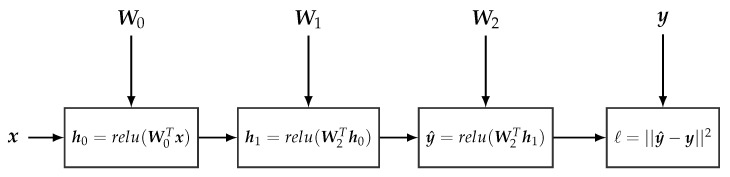
Example of a simple computational graph encoding a neural network with two hidden layers and the squared loss function. Note that each operation node encapsulates a part of the CG encoding the associated operations, we do not expand the whole CG for the sake of simplicity.

**Example** **5.** *Figure 4 provides an example of a computational graph encoding a neural network with x as input, $\hat{y}$ as output, and two hidden layers. This computational graph also encodes the loss function $\ell (y,\hat{y})$. As computational graphs can be defined over tensors, the above computational graph can encode the forward (and backward) pass of the neural network for a whole data batch x, and thereby also provide the loss (and the gradient) for this set of data samples. Algorithm 2 shows the pseudo-code description for defining and learning this neural network using standard gradient descent.*

**Algorithm 2** Pseudo-code of the definition and learning of a simple neural network.**input*x***, ***y*** the labels.
 # Define the computational graph encoding the ANN and the loss function $W_{0},W_{1},W_{2}=Parameters\left(\right)$
 $h_{0}=relu\left(W_{0}^{T}x\right)$
 $h_{1}=relu\left(W_{1}^{T}h_{0}\right)$
 $\hat{y}=relu\left(W_{2}^{T}h_{1}\right)$
 *ℓ* = $\left|\right|\hat{y}-y{\left|\right|}^{2}$ # Follow the gradients until convergence. $W=(W_{0},W_{1},W_{2})$
 **repeat**
  $W=W-\rho \nabla_{W}\ell$
 **until** convergence


## 4. Probabilistic Models with Deep Neural Networks

### 4.1. Deep Latent Variable Models

LVMs have usually been restricted to the conjugate exponential family because, in this case, inference is feasible (and scalable) as we showed in Section 2. But recent advances in VI (which will be outline in Section 5) have enabled LVMs to be extended with DNNs. Variational auto-encoders (VAE) [23,59] are probably the most influential models combining LVMs and DNNs. VAEs extend the classical technique of PCA for data representation in lower-dimensional spaces. More precisely, the probabilistic version of the PCA model [41] is extended in [23], where the relationship between the low-dimensional representation and the observed data is governed by a DNN, i.e., a highly non-linear function, as opposed to the standard linear transformation in the basic version of the PCA model. These models are able to capture more compact low-dimensional representations, especially in cases where data is high-dimensional but “lives” in a low-dimensional manifold [60]. This is, e.g., the case for image data [23,61,62,63,64], text data [65], audio data [66], chemical molecules [67], to name some representative applications of this technique. We note that, in this section and in the following ones, we will use VAEs as a running example illustrating how DNNs can be used in probabilistic modeling.

VAEs have also given rise to a plethora of extensions of classic LVMs to their *deep* counterpart. For instance, different examples of this approach are given in [68], along with extensions of Gaussian mixture models, latent linear dynamical systems and latent switching linear dynamical systems with the non-linear relationships modeled by DNNs. Hidden semi-Markov models are extended with recurrent neural networks in [69]. Extensions of popular LDA models [40] for uncovering topics in text data can be found in [70,71]. Many other works are following the same trend [72,73,74,75].

**Example** **6.** *VAEs are widely adopted LVMs containing DNNs [23]. Algorithm 3 provides a simplified pseudo-code description of the generative part of a VAE model. It can also be seen as a non-linear probabilistic PCA model, where the non-linearity is included in the form of an artificial neural network.*

*This model is quite similar to the PCA model presented in Example 1. The main difference comes from the conditional distribution of X. In the PCA model, the mean of the normal distribution of X linearly depends on Z through β. In the VAE model, the mean depends on Z through a DNN parametrized by β. This DNN is also known as the decoder network of the VAE [23].*

*Note that some formulations of this model also include another DNN component, which connects Z with the variance $\sigma^{2}$ of the normal distribution of X; for the sake of simplicity, we have not included this extension in the example.*

**Figure 5:**
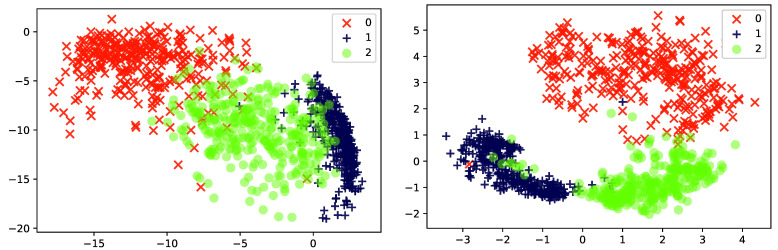
Two-dimensional latent representations of the the MNIST dataset resulting of applying: (**Left**) a standard probabilistic PCA (reproduced from Figure 2 to ease comparison), and (**Right**) a non-linear probabilistic PCA with a ANN containing a hidden layer of size 100 with a *relu* activation function.

*Figure 5 experimentally illustrates the advantage of using a non-linear PCA model over the classic PCA model. As can be seen, the non-linear version separates more clearly the three digits than the linear model did. We shall return to this example in Section 5.2, where we will introduce the so-called encoder network used for inference.*

**Algorithm 3** Pseudo-code of the generative model of a variational auto-encoder (or non-linear probabilistic PCA).
# Define the global parameters
$\alpha_{0},\beta_{0},\alpha_{1},\beta_{1}∼N\left(0,I\right)$

**for** i=1,…,N **do**
 # Define the local latent variables $Z_{i}∼N\left(0,I\right)$
 # Define the ANN with a single hidden layer $h_{i}$ $h_{i}=relu\left(\beta_{0}^{T}z_{i}+\alpha_{0}\right)$
 $\mu_{i}=\beta_{1}^{T}h_{i}+\alpha_{1}$
 # Define the observed variables $X_{i}∼N\left(\mu_{i},\sigma^{2}I\right)$

**end for**



LVMs with DNNs can also be found in the literature under the name of deep generative models [25,26,27,28]. They generate data samples using probabilistic constructs that include DNNs. This new capacity has also resulted in substantial impact within the deep learning community because it has opened up for the possibility of dealing with unsupervised learning problems, e.g., in the form of generative adversarial nets [27]. This should be seen in contrast to the classic deep learning methods, which are mainly focused on supervised learning settings. In any case, this active area of research is out of the scope of this paper and contains many alternative models, which do not fall within the category of the models explored in this paper (Ref. [76] provides a recent survey of this field).

### 4.2. Stochastic Computational Graphs

**Figure 6:**
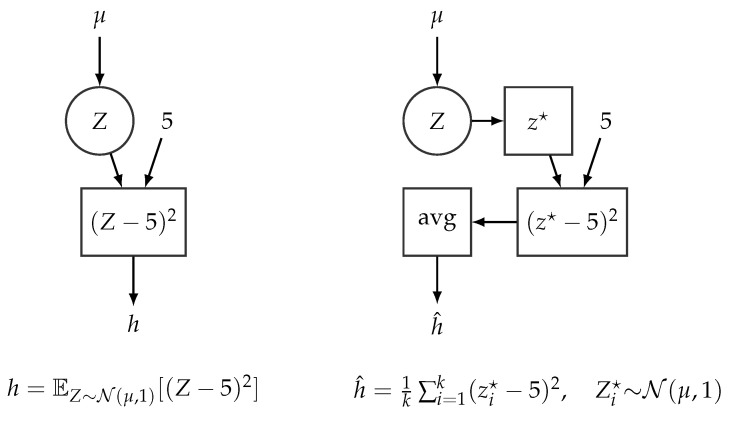
(**Left**) A stochastic computational graph encoding the function $h=E_{Z}\left[{\left(Z-5\right)}^{2}\right]$, where Z∼N(μ,1). (**Right**) Computational graph processing *k* samples from *Z* and producing $\hat{h}$, an estimate of $E_{Z}\left[{\left(Z-5\right)}^{2}\right]$.

The key data structure for representing probabilistic models with deep neural networks is the so-called stochastic computational graph (SCG) [77]. SCGs extend standard computational graphs (defined Section 3.2 with stochastic nodes (represented as circles in the subsequent diagrams). The probability distributions associated with stochastic nodes are defined conditionally on their parents and enable the specification of complex functions involving expectations over random variables. Figure 6 (**Left**) shows an example of a simple SCG involving an expectation over a random variable Z. Modern PPLs support a wide and diverse range of probability distributions for defining SCGs [78]. These probability distributions are defined over tensor objects to seamlessly accommodate the underlying CGs, which define operations over tensor objects too.

We note that SCGs are not directly implemented within PPLs, because computing the exact expected value of a complex function is typically infeasible. However, they are indirectly included through the use of a standard computational graph engine: Each stochastic node, Z, is associated with a tensor, $z^{⋆}$, which represents a (set of) sample(s) from the distribution associated with z, and the generated samples can thus be fed to the underlying computational graph through the tensor $z^{⋆}$. Hence, SCGs can be *simulated* by sampling from the stochastic nodes and processing these samples by a standard CG. Figure 6 illustrates how SCG can be simulated using standard CGs. Note that CGs are designed to operate efficiently with tensors (current toolboxes like TensorFlow exploit high-performance computing hardware such as GPUs or TPUs [34]), and it is therefore much more efficient to run the CG once over a collection of samples, rather than running the CG multiple times over a single sample.

**Figure 7:**
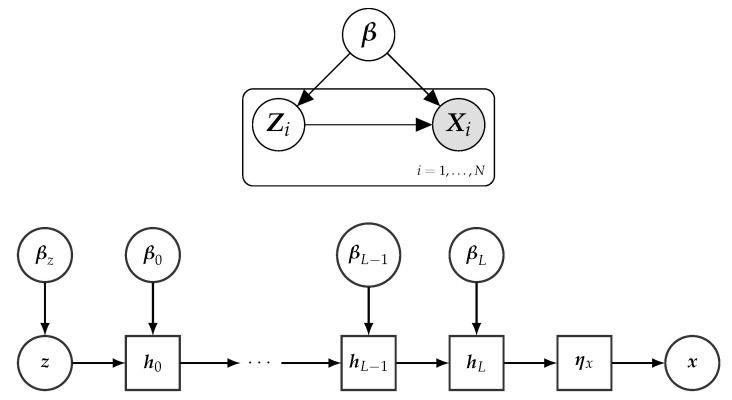
The top part depicts a probabilistic graphical model using plate notation [8]. The lower part depicts an abstract representation of a stochastic computational graph encoding the model, where the relation between z and x is defined by a DNN with L+1 layers. See Section 4 for details.

In this way, SCGs can be used to define and support inference and learning of general probabilistic models, including the ones referenced in Section 4.1. More generally, all the concepts outlined in this paper apply to any probabilistic model that can be defined by means of an SCG or which can be compiled into an equivalent SCG representation. For instance, the following model specification (illustrated by the top part in Figure 7) relating Z with the natural parameters $\eta_{x}$ of x can be equivalently represented by the SCG illustrated in the lower part of Figure 7.

(16) $$\begin{array}{ccc} \ln p(\beta ) & = & \ln h\left(\beta \right)+\alpha^{T}t\left(\beta \right)-a_{g}\left(\alpha \right),\\ \ln p(z_{i}|\beta ) & = & \ln h\left(z_{i}\right)+\eta_{z}{\left(\beta \right)}^{T}t\left(z_{i}\right)-a_{z}\left(\eta_{z}\left(\beta \right)\right),\\ h_{0} & = & r_{0}\left(z_{i}^{T}\beta_{0}\right),\\ & \ldots & \\ h_{l} & = & r_{l}\left(h_{l-1}^{T}\beta_{l-1}\right),\\ & \ldots & \\ h_{L} & = & r_{L}\left(h_{L}^{T}\beta_{L}\right),\\ \ln p(x_{i}|z_{i},\beta ) & = & \ln h\left(x_{i}\right)+\eta_{x}{\left(h_{L}\right)}^{T}t\left(x_{i}\right)-a_{x}\left(\eta_{x}\left(h_{L}\right)\right). \end{array}$$

From this example, we again see the main difference with respect to standard LVMs (see Section 2.1) is the conditional distribution of the observations $x_{i}$ given the local hidden variables $Z_{i}$ and the global parameters β, which is here governed by a DNN parameterized by β.

## 5. Variational Inference with Deep Neural Networks

Similarly to standard probabilistic models, performing variational inference in deep latent variable models (as described in the previous section) also reduces to maximizing the ELBO function L(λ,ϕ) given in Section 2.2 (Equation (8)); recall that this is equivalent to minimizing the KL divergence between the variational posterior q(β,z|λ,ϕ) and the target distribution p(β,z|x). However, as was also noted in the previous section, when the probabilistic model contains complex constructs like DNNs, it falls outside the conjugate exponential family and the traditional VI methods, tailored to this specific family form, can therefore not be applied.

In terms of the variational distribution, we will still assume the same factorization scheme defined in Equation (5) for the deep latent variable models considered in this section. However, as we will see below, we need not adopt the conjugate models’ strong restrictions on the variational approximation family (see Equation (6)). Instead, the only (and much weaker) restriction that we will impose is that (*i*) the log probability of the variational distribution, lnq(β,z|λ,ϕ), can be represented by a computational graph (and, as a consequence, that it is differentiable wrt. λ and ϕ) and (ii) that we can sample from the variational distribution q(β,z|λ,ϕ). Depending on the specific method being applied, additional requirements may be introduced.

In the rest of the section, we will give an overview of the two main techniques employed in modern variational methods to perform inference in probabilistic models with deep neural networks. In Section 5.1, we provide an overview to black-box variational inference [24,79,80] whose main purpose is the computation of the gradient of the ELBO. Within this section, we described the two main approaches for computing this gradient using Monte Carlo methods. Section 5.2 introduces amortized variational inference [81,82], a widely used technique for dealing with probabilistic models containing local latent variables [23]. We end this section by discussing the pros and cons of each of the presented methods.

### 5.1. Black Box Variational Inference

**Figure 8:**
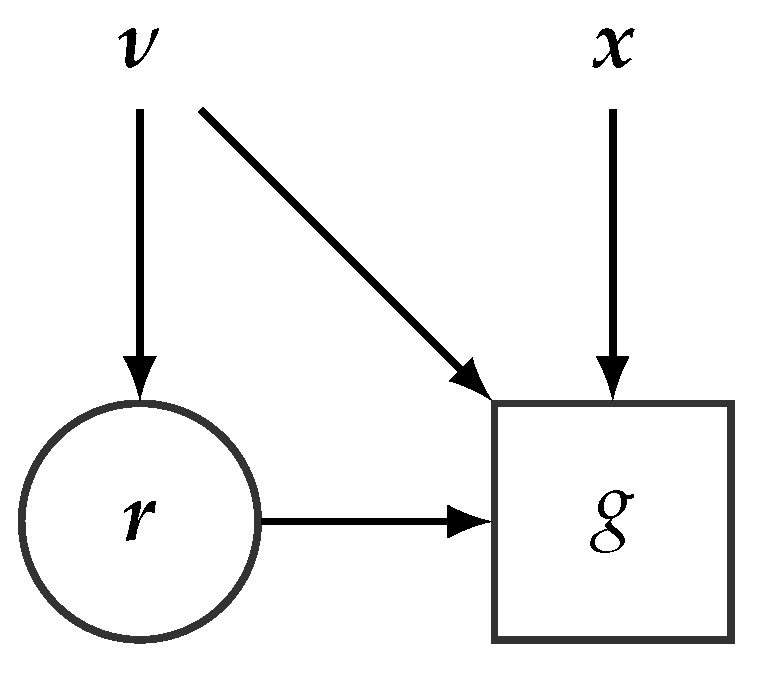
SCG representing the ELBO function L(ν). r is distributed according to the variational distribution, r∼q(r|ν).

For the sake of presentation, we reparameterize the ELBO function with r=(β,z) and ν=(λ,ϕ) and define g(r,ν)=lnp(x,r)−lnq(r|ν). With this notation the ELBO function L of Equation (8) can then be expressed as

(17) $$L\left(\nu \right)=E_{R}\left[g\left(r,\nu \right)\right]=\int q\left(r|\nu \right)g\left(r,\nu \right)dr,$$

from which we see that the ELBO function can easily be represented by an SCG as shown in Figure 8. If the SCG in Figure 8 did not include stochastic nodes (thus corresponding to a standard CG), we could, in principle, perform variational inference (maximizing L(ν) wrt. ν) by simply relying on automatic differentiation and a variation of gradient ascent. However, optimizing over SCGs is much more challenging because automatic differentiation does not readily apply. The problem is that the variational parameters ν (wrt. which we should differentiate) also affects the expectation inherent in the ELBO function, see Equation (17):

(18) $$\nabla_{\nu }L=\nabla_{\nu }E_{R}\left[g\left(r,\nu \right)\right].$$

In the case of conjugate models, we can take advantage of their properties and derive closed-form solutions for this problem, as detailed in Section 2.2. In general, though, there are no closed-form solutions for computing gradients in non-conjugate models; a simple concrete example is the Bayesian logistic regression model [6] (p. 756).

In this section, we provide two generic solutions for computing the gradient of the ELBO function for probabilistic models including DNNs. Both methods directly rely on the automatic differentiation engines available for standard computational graphs. In this way, the methods can be seen as extending the automatic differentiation methods of standard computational graphs to SCGs, giving rise to a powerful approach to VI for generic probabilistic models. The main idea underlying both approaches is to compute the gradient of the expectation given in Equation (18) using Monte Carlo techniques. More precisely, we will show how we can build unbiased estimates of this gradient by sampling from the variational (or an auxiliary) distribution without having to compute the gradient of the ELBO analytically [24,79,80].

#### 5.1.1. Pathwise Gradients

The idea of this approach is to exploit reparameterizations of the variational distribution in terms of deterministic transformations of a noise distribution [83,84]. A distribution q(r|ν) is reparameterizable if it can be expressed as

(19) $$\begin{array}{cc} \; & \epsilon ∼q(\epsilon ),\\ \; & r=t(\epsilon ;\nu ), \end{array}$$

where ϵ does not depend on parameter ν and t(·;ν) is a deterministic function which encapsulates the dependence of r with respect to ν. This transforms the expectation over r to an expectation over ϵ. By exploiting this reparameterization property, we can express the gradient of L in Equation (18) as [23,85,86],

(20) $$\begin{array}{cc} \nabla_{\nu }L\left(\nu \right) & =\nabla_{\nu }E_{R}\left[g(r,\nu )\right]\\ \; & =\nabla_{\nu }E_{\epsilon }\left[g(t(\epsilon ;\nu ),\nu )\right]\\ \; & =E_{\epsilon }\left[\nabla_{\nu }g\left(t\left(\epsilon ;\nu \right),\nu \right)\right]\\ \; & =E_{\epsilon }\left[\nabla_{t}g{\left(t\left(\epsilon ;\nu \right),\nu \right)}^{T}\nabla_{\nu }t\left(\epsilon ;\nu \right)+\nabla_{\nu }g\left(t\left(\epsilon ;\nu \right),\nu \right)\right]\\ \; & =E_{\epsilon }\left[\nabla_{r}g{\left(r,\nu \right)}^{T}\nabla_{\nu }t\left(\epsilon ;\nu \right)+\nabla_{\nu }g\left(r,\nu \right)\right]\\ \; & =E_{\epsilon }\left[\nabla_{r}g{\left(r,\nu \right)}^{T}\nabla_{\nu }t\left(\epsilon ;\nu \right)\right]. \end{array}$$

In the last step we have exploited that $E_{\epsilon }\left[\nabla_{\nu }g\left(r,\nu \right)\right]=0$. To see this, we first utilize that

$$E_{\epsilon }\left[\nabla_{\nu }g\left(r,\nu \right)\right]=\int q\left(\epsilon \right)\nabla_{\nu }g\left(r,\nu \right)d\epsilon =\int q\left(r|\nu \right)\nabla_{\nu }g\left(r,\nu \right)dr=E_{R}\left[\nabla_{\nu }g\left(r,\nu \right)\right].$$

Next, as g(r,ν)=lnp(x,r)−lnq(r|ν), it follows that $\nabla_{\nu }g\left(r,\nu \right)=-\nabla_{\nu }\ln q\left(r|\nu \right)$. Finally, since $E_{R}\left[\nabla_{\nu }\ln q\left(r|\nu \right)\right]=0$, we have that $E_{\epsilon }\left[\nabla_{\nu }g\left(r,\nu \right)\right]=0$.

Note that once we employ this reparameterization trick, the gradient enters the expectation, and afterwards we simply apply the chain rule of derivatives. Here, it is also worth noticing that the gradient estimator is informed by the gradient with respect to g, which gives the direction of the maximum posterior mode (we shall return to this issue in Section 5.1.2).

**Example** **7.** *The normal distribution is the best known example where this technique can be applied: a variable $W∼N(\mu ,\sigma^{2})$ can be reparameterized as ϵ∼N(0,1) and W=μ+σϵ. So, by exploiting this reparameterization, we can compute the gradient of stochastic functions as the one defined in Figure 6, i.e., compute $\nabla_{\mu }E_{Z}\left[{\left(Z-5\right)}^{2}\right]$, where Z∼N(μ,1),*

$$\nabla_{\mu }E_{Z}\left[{\left(Z-5\right)}^{2}\right]=E_{\epsilon }\left[\nabla_{\mu }{\left(\mu +\epsilon -5\right)}^{2}\right]=E_{\epsilon }\left[2(\mu +\epsilon -5)\right]=2\left(\mu -5\right).$$

*In practice, this expectation is approximated using Monte Carlo sampling,*

$$\nabla_{\mu }E_{Z}\left[{\left(Z-5\right)}^{2}\right]\approx \frac{1}{K}\sum_{i=1}^{K}2\left(\mu +\epsilon_{i}-5\right)\;\epsilon_{i}∼N\left(0,1\right).$$

**Figure 9:**
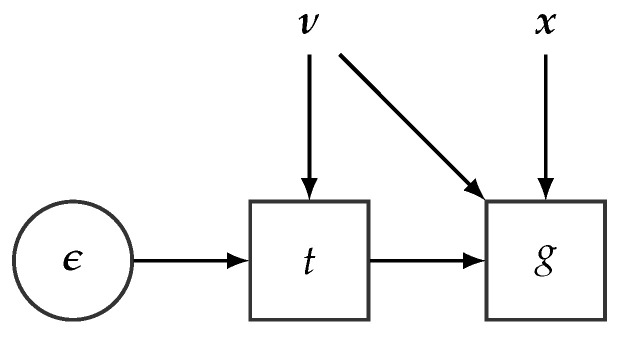
Reparameterized SCG representing the ELBO function L(ν).

In terms of SCGs, this reparameterization trick can be captured by the transformation of the (original) SCG shown in Figure 8 to the SCG shown in Figure 9. For the transformed SCG, the underlying CG (exemplified in Figure 6) can be readily applied and from automatic differentiation we obtain unbiased estimates of the gradients of the ELBO.

More generally, and pertinently, through the reparameterization trick we can define a CG representation of the ELBO function L, which in turn can be used for computing a Monte Carlo estimation of L,

(21) $$\hat{L}=\frac{1}{K}\sum_{i=1}^{K}\ln p\left(x,t\left(\epsilon_{i};\nu \right)\right)-\ln q\left(t\left(\epsilon_{i};\nu \right)|\nu \right)\;\epsilon_{i}∼q\left(\epsilon \right),$$

and the associated automatic differentiation engine of the CG can be used for finding the derivatives of L (cf. Equation (20)). The CG thus also serves as a generic tool for abstracting away and hiding the details of the gradient calculations from the user.

The applicability of the reparameterization trick only extends to distributions that can be expressed in the form shown in Equation (19). Fortunately, [87] recently introduced an implicit reparameterization approach, which apply to a wider range of distributions including Gamma, Beta, Dirichlet and von Mises (i.e., distributions not covered by Equation (19)). This method computes the gradient of L as

(22) $$\nabla_{\nu }L\left(\nu \right)=-E_{R}\left[\frac{\nabla_{r}g{\left(r,\nu \right)}^{T}\nabla_{\nu }F\left(r;\nu \right)}{q(r|\nu )}\right],$$

where F(r;ν) is the cumulative distribution function of q(r|ν). Other similar approaches have been proposed for models with discrete latent random variables [88,89].

The above family of gradient estimators usually have lower variance than other methods [44] and, in many cases, they can even provide good estimates with a single Monte Carlo sample. However, the estimators only apply to distributions that support explicit or implicit reparameterizations. Although many distributions provide this support, there are also other relevant distributions, such as the multinomial distribution, which cannot be handled using either of the reparameterization techniques.

**Example** **8.** *We end this sub-section with our running example about VAEs. In this case, we consider a VAE without an encoder network; the encoder network will be discussed in the Section 5.2. This model can thus be seen as a non-linear PCA model (the non-linearity is defined in terms of an ANN) as described in Example 6. For this model, the ELBO function can be expressed as*

$$\begin{array}{ccc} L(\lambda ,\phi ) & = & E_{q}\left[\ln p\left(x|z,\beta \right)\right]+E_{q}\left[\ln p\left(z\right)\right]+E_{q}\left[\ln p\left(\beta \right)\right]\\ & & -E_{q}[\ln q\left(z|\phi \right)-E_{q}\left[\ln q\left(\beta |\lambda \right)\right]. \end{array}$$

*Algorithm 4 gives a pseudo-code specification of the SCG defining the ELBO function using the reparameterization trick; here we only use a single sample from the variational distribution q(β,z|λ,ϕ) in reparameterized form. The definition of the ELBO function L is introduced together with the specification of the decoder network, hence gradients wrt. the variational parameters can be readily computed and optimized using standard algorithms.*

**Algorithm 4** Pseudo-code for defining the ELBO function $\hat{L}$, and by translation the SCG, of a VAE with no encoder network (see Algorithm 3). We use a single sample to compute the Monte Carlo estimate of $\hat{L}$ (see Equation (21)). $\ln p_{N}\left(\cdot |\cdot ,\cdot \right)$ denotes the log-probability function of a normal distribution.**input** Data: ***x****_train_*, Variational Parameters: λ,ϕ
 # Sample (using reparameterization) from q(β|λ) and q(z|ϕ). $\epsilon_{\alpha_{0}},\epsilon_{\beta_{0}},\epsilon_{\alpha_{1}},\epsilon_{\beta_{1}},\epsilon_{z}∼N\left(0,I\right)$
 $\alpha_{0}=\lambda_{\alpha_{0},\mu }+\epsilon_{\alpha_{0}}\lambda_{\alpha_{0},\sigma },\;\beta_{0}=\lambda_{\beta_{0},\mu }+\epsilon_{\beta_{0}}\lambda_{\beta_{0},\sigma }$
 $\alpha_{1}=\lambda_{\alpha_{1},\mu }+\epsilon_{\alpha_{1}}\lambda_{\alpha_{1},\sigma },\;\beta_{1}=\lambda_{\beta_{1},\mu }+\epsilon_{\beta_{1}}\lambda_{\beta_{1},\sigma }$
 $z=\phi_{z,\mu }+\epsilon_{z}\phi_{z,\sigma }$
 # Pass the variational sample z through the decoder ANN $h_{0}=relu\left(z\beta_{0}^{T}+\alpha_{0}\right)$
 $\mu_{x}=h_{0}\beta_{1}^{T}+\alpha_{1}$
 # Define the “energy part” of the ELBO function $E_{q}\left[\ln p\left(x_{train},z,\alpha ,\beta \right)\right]$. $L=\ln p_{N}\left(x_{train}|\mu_{x},\sigma_{x}^{2}I\right)$
 $L=L+\ln p_{N}\left(z|0,I\right)+\sum_{i}\ln p_{N}\left(\alpha_{i}|0,I\right)+\ln p_{N}\left(\beta_{i}|0,I\right)$
 # Define the “entropy part” of the ELBO function $E_{q}\left[\ln q\left(z,\alpha ,\beta \right)\right]$. $L=L-\ln p_{N}\left(z|\phi_{z,\mu },\phi_{z,\sigma }^{2}\right)$
 $L=L-\sum_{i}\ln p_{N}\left(\alpha_{i}|\lambda_{\alpha_{i},\mu },\lambda_{\alpha_{i},\sigma }^{2}\right)-\sum_{i}\ln p_{N}\left(\beta_{i}|\lambda_{\beta_{i},\mu },\lambda_{\beta_{i},\sigma }^{2}\right)$
 return L


#### 5.1.2. Score Function Gradients

This is a classical method for gradient estimation, also known as the REINFORCE method [24,90,91]. It builds on the following generic transformations to compute the gradient of an expected value,

(23) $$\begin{array}{cc} \nabla_{\nu }L\left(\nu \right) & =\nabla_{\nu }\int q\left(r|\nu \right)g\left(r,\nu \right)dr\\ \; & =\int g\left(r,\nu \right)\nabla_{\nu }q\left(r|\nu \right)+q\left(r|\nu \right)\nabla_{\nu }g\left(r,\nu \right)dr\\ \; & =\int g\left(r,\nu \right)\;q\left(r|\nu \right)\nabla_{\nu }\ln q\left(r|\nu \right)+q\left(r|\nu \right)\nabla_{\nu }g\left(r,\nu \right)dr\\ \; & =E_{R}\left[g\left(r,\nu \right)\;\nabla_{\nu }\ln q\left(r|\nu \right)+\nabla_{\nu }g\left(r,\nu \right)\right]. \end{array}$$

Following the discussion surrounding the derivation of Equation (20), we have that $E_{R}\left[\nabla_{\nu }g\left(r,\nu \right)\right]=E_{R}\left[-\nabla_{\nu }\ln q\left(r|\nu \right)\right]=0$ and the gradient of the ELBO therefore simplifies to

(24) $$\nabla_{\nu }L\left(\nu \right)=E_{R}\left[g\left(r,\nu \right)\;\nabla_{\nu }\ln q\left(r|\nu \right)\right].$$

The term $\nabla_{\nu }\ln q\left(r|\nu \right)$ (the gradient of the log of a probability distribution) is referred to as the score function, hence the name of the method.

From the above equation, we obtain unbiased estimates of the gradient by sampling from q(r|ν). This method is general in the sense that it only requires being able to evaluate the function g(r,ν) and computing the score function, $\nabla_{\nu }\ln q\left(r|\nu \right)$. In consequence, the method applies to a wide range of models, including those covered by the pathwise gradient estimator. However, in practice, the score function gradient often yields high variance estimates when the dimensionality of ν is relatively high. This is accentuated by the gradient estimator only being guided by the gradient of the (log of the) variational distribution and not the likelihood term of the model (which was the case for the pathwise gradient estimator). To reduce the variance, one often relies on variance reduction techniques for improved performance [24,86,92,93], but, still, in a practical setting the score function estimator mostly serve as the fall-back method when the pathwise gradient estimator is not applicable.

**Example** **9.** *We revisit Example 7. We have to compute the gradient of an expectation $\nabla_{\mu }E_{Z}\left[{\left(Z-5\right)}^{2}\right]$, where Z∼N(μ,1). By applying the score function gradient estimator, we get*

$$\begin{array}{ccc} \nabla_{\mu }E_{Z}\left[{\left(Z-5\right)}^{2}\right] & = & E_{Z}\left[{\left(Z-5\right)}^{2}\nabla_{\mu }\ln N\left(Z|\mu ,1\right)\right]\\ & = & E_{Z}\left[{\left(Z-5\right)}^{2}\nabla_{\mu }\left(-\frac{1}{2}{\left(Z-\mu \right)}^{2}\right)\right]\\ & = & E_{Z}\left[{\left(Z-5\right)}^{2}\left(Z-\mu \right)\right], \end{array}$$

*which can be approximated by Monte Carlo sampling, $\nabla_{\mu }E_{Z}\left[{\left(Z-5\right)}^{2}\right]\approx \frac{1}{K}\sum_{i=1}^{K}{\left(z_{i}-5\right)}^{2}\left(z_{i}-\mu \right)$, where $z_{i}$ are samples from N(μ,1).*

In [94], it is detailed an elegant implementation of this technique using SCGs.

### 5.2. ELBO Optimization with Amortized Variational Inference

In principle, we can address the optimization of the ELBO function using an off-the-shelf gradient ascent algorithm combined with the techniques presented in the previous section. The ELBO function L(λ,ϕ), in this case, is again expressed in terms of global variational parameters λ (defining the variational distribution over the global latent variables q(β|λ)) and in terms of local variational parameters ϕ (defining the variational distribution over the local latent variables $q(z_{i}|\phi_{i})$); we implicitly assume that the variational posterior fully factorizes, as shown in Equation (5), although this assumption is not crucial for the discussion below. Unfortunately, as the number of local variational parameters $\phi =(\phi_{1},\ldots ,\phi_{N})$ grows with the size *N* of the data set, straight-forward optimization using gradient ascent quickly becomes computationally infeasible as the size of the data grows.

To address this issue we can rely on some of the tricks detailed in Section 2.3. First, we can express L(λ,ϕ) only in terms of λ, as previously shown in Equations (11) and (12),

$$L\left(\lambda \right)=E_{q}\left[\ln p\left(\beta \right)\right]-E_{q}\left[\ln q\left(\beta |\lambda \right)\right]+\sum_{i=1}^{N}\max_{\phi_{i}}\left(E_{q}\left[\ln p\left(x_{i},Z_{i}|\beta \right)\right]-E_{q}\left[\ln q\left(Z_{i}|\phi_{i}\right)\right]\right).$$

As done in Section 2.3, we can get unbiased noisy estimates of this ELBO by data subsampling. If *I* is a randomly chosen data index, I∈{1,…,N}, and

$$L_{I}\left(\lambda \right)=E_{q}\left[\ln p\left(\beta \right)\right]-E_{q}\left[\ln q\left(\beta |\lambda \right)\right]+N\max_{\phi_{I}}\left(E_{q}\left[\ln p\left(x_{I},Z_{I}|\beta \right)\right]-E_{q}\left[\ln q\left(Z_{I}|\phi_{I}\right)\right]\right),$$

then the expectation of $L_{I}\left(\lambda \right)$ is equal to L(λ) [19] and computing the gradient of $L_{I}\left(\lambda \right)$ wrt. λ will give us a noisy unbiased estimate. However, in this case, we require solving an maximization problem for each subsampled data point (i.e., $\max_{\phi_{I}}$). In the case of conjugate exponential models, this inner maximization step can be computed in closed form as shown in Equation (10). However, for models outside the conjugate exponential family, we would have to resort to iterative algorithms, based on the methods described in Section 5.1, making the approach infeasible.

Amortized inference [81,82] aims to address this problem by learning a mapping function, denoted by s, between $x_{i}$ and $\phi_{i}$ parameterized by θ, i.e., $\phi_{i}=s\left(x_{i}|\theta \right)$. Hence, $L_{I}\left(\lambda \right)$ is expressed as $L_{I}\left(\lambda ,\theta \right)$,

$$\begin{array}{ccc} L_{I}\left(\lambda ,\theta \right) & = & E_{q}\left[\ln p\left(\beta \right)\right]-E_{q}\left[\ln q\left(\beta |\lambda \right)\right]\\ & + & N\cdot E_{q}\left[\ln p\left(x_{I},Z_{I}|\beta \right)\right]-N\cdot E_{q}\left[\ln q\left(Z_{I}|x_{I},\phi_{I}=s\left(x_{i}|\theta \right)\right)\right]. \end{array}$$

The parameter vector θ is shared among all the data points and does not grow with the data set as was previously the case when each data point was assigned its own local variational parameters, $\phi =\{\phi_{1},\ldots ,\phi_{N}\}$. On the other hand, amortized inference assumes that the parameterized function s is flexible enough to allow for the estimation of the local variational parameters $\phi_{i}$ from the data points $x_{i}$. Thus, the family of variational distributions defined by this technique,

$$q\left(\beta ,z|x,\lambda ,\theta \right)=q\left(\beta |\lambda \right)\prod_{i=1}^{N}q\left(z_{i}|x_{i},\phi_{i}=s\left(x_{i}|\theta \right)\right),$$

is more restricted than the one defined in Equation (5), which directly depends of λ and ϕ. So, there is a trade-off between flexibility in the variational approximation and computational efficiency when applying amortized inference techniques.

Note that the amortized function greatly simplifies the use of the model when making predictions over unseen data $x^{\prime }$. If we need the posterior $p(z^{\prime }|x^{\prime })$ over a new data sample $x^{\prime }$ (e.g., for dimensionality reduction when using a VAE model), we just need to invoke the learnt amortized inference function to recover this posterior, $q(z^{\prime }|\phi =s\left(x^{\prime }|\theta^{⋆}\right))$.

An unbiased estimate of the gradient of $L_{I}\left(\lambda ,\theta \right)$ wrt to λ and θ can be computed using the techniques described in the previous section, as both affect an expectation term. Note that the unbiased estimate of the gradient of $L_{I}\left(\lambda ,\theta \right)$ is also an unbiased estimate of the gradient of L(λ,θ). Similar to Equation (13), the ELBO can be maximized by following noisy estimates of the gradient,

(25) $$\begin{array}{c} \lambda_{t+1}=\lambda_{t}+\rho_{t}{\hat{\nabla }}_{\lambda }L_{I_{t}}\left(\lambda_{t},\theta_{t}\right),\\ \theta_{t+1}=\theta_{t}+\rho_{t}{\hat{\nabla }}_{\theta }L_{I_{t}}\left(\lambda_{t},\theta_{t}\right) \end{array}$$

where $I_{t}$ are the indexes of randomly subsampled data points at time step *t*.

**Example** **10.** *We finally arrive at the original formulation of VAEs, which includes an amortized inference function linking the data samples with the latent variables Z. This amortized function takes the form of a neural network and is referred to as the encoder network because it translates an observation X to (a distribution over) its hidden representation Z; recall that the decoder network (part of the model specification) links the latent variables Z to (a distribution over) the observable variables X. The existence of these two networks, the encoder and the decoder, establishes a direct link with the previously known auto-encoder networks [95]. In this example, both the encoder and the decoder network have a single hidden layer with a relu activation function.*

*Algorithm 5 shows pseudo-code defining the ELBO function associated with this model. The model falls outside the conjugate exponential family, but due to distributional assumptions of the VAE’s we can estimate the gradients by applying the reparameterization trick (see Section 5.1.1). Specifically, from the encoder network, we sample from the variational distribution over $Z_{I}$ given $X_{I}$ (in reparameterized form), and at the end of the algorithm, we define the ELBO function $L_{I}$, which includes the definition of the decoder network. As for the previous example, the pseudo-code specification directly translates into a computational graph. From this representation, the gradients wrt. the variational parameters can be readily computed and the ELBO function optimized using, in this case, stochastic gradient ascent or some of a variation hereof.*

**Figure 10:**
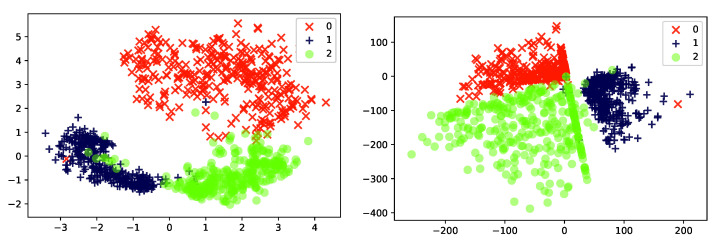
Two-dimensional latent representation of the the MNIST dataset resulting of applying: (**Left**) a non-linear probabilistic PCA, and (**Right**) a VAE. The ANNs of the non-linear PCA and the ones defining the VAE’s decoder and encoder contain a single hidden layer of size 100.

* Figure 10 shows the two-dimensional latent embedding found by the non-linear probabilistic PCA (Left; reproduced from  Figure 5) and VAE (Right) for the same reduced MNIST data set used previously. The three classes are clearly separated in latent space for both models.*

**Algorithm 5** Pseudo-code for the estimation of the ELBO function $L_{I}$ of a Variational Auto-encoder. We use a single sample to compute the Monte Carlo estimation of $\hat{L}$ (see Equation (21)). $\ln p_{N}\left(\cdot |\cdot ,\cdot \right)$ denotes the log-probability function of a Normal distribution.**input** Data: ***x****_I_* a single data-sample, *N* size of the data, Variational Parameters: λ,θ
 # Sample (using reparameterization) from q(β|λ). $\epsilon_{\theta_{0}},\epsilon_{\theta_{0}^{\prime }},\epsilon_{\theta_{1}},\epsilon_{\theta_{1}^{\prime }}∼N\left(0,I\right)$
 $\theta_{0}=\lambda_{\theta_{0},\mu }+\epsilon_{\theta_{0}}\lambda_{\theta_{0},\sigma },\;\theta_{0}^{\prime }=\lambda_{\theta_{0}^{\prime },\mu }+\epsilon_{\theta_{0}^{\prime }}\lambda_{\theta_{0}^{\prime },\sigma }$
 $\theta_{1}=\lambda_{\theta_{1},\mu }+\epsilon_{\theta_{1}}\lambda_{\theta_{1},\sigma },\;\theta_{1}^{\prime }=\lambda_{\theta_{1}^{\prime },\mu }+\epsilon_{\theta_{1}^{\prime }}\lambda_{\theta_{1}^{\prime },\sigma }$
 # Pass x through the encoder network and sample $z_{I}∼q\left(z|\phi =s\left(x_{I}|\theta \right)\right)$ $h_{z,0}=relu\left(x_{I}\theta_{0}^{T}+\theta_{0}^{\prime }\right)$
 $h_{z,1}=h_{z,0}\theta_{1}^{T}+\theta_{1}^{\prime }$
 # $h_{z,1}$ contains both the mean, $h_{z,1,\mu }$, and the scale, $h_{z,1,\sigma }$. $\epsilon_{z}∼N\left(0,I\right)$
 $z_{I}=h_{z,1,\mu }+\epsilon_{z}h_{z,1,\sigma }$
 # Pass the variational sample z through the decoder network $\epsilon_{\alpha_{0}},\epsilon_{\beta_{0}},\epsilon_{\alpha_{1}},\epsilon_{\beta_{1}}∼N\left(0,I\right)$
 $\alpha_{0}=\lambda_{\alpha_{0},\mu }+\epsilon_{\alpha_{0}}\lambda_{\alpha_{0},\sigma },\;\beta_{0}=\lambda_{\beta_{0},\mu }+\epsilon_{\beta_{0}}\lambda_{\beta_{0},\sigma }$
 $\alpha_{1}=\lambda_{\alpha_{1},\mu }+\epsilon_{\alpha_{1}}\lambda_{\alpha_{1},\sigma },\;\beta_{1}=\lambda_{\beta_{1},\mu }+\epsilon_{\beta_{1}}\lambda_{\beta_{1},\sigma }$
 $h_{0}=relu\left(z_{I}\beta_{0}^{T}+\alpha_{0}\right)$
 $\mu_{x}=h_{0}\beta_{1}^{T}+\alpha_{1}$
 # Define the “energy part” of the ELBO function $L_{I}=N\cdot \ln p_{N}\left(x_{I}|\mu_{x},\sigma_{x}^{2}I\right)+N\cdot \ln p_{N}\left(z_{I}|0,I\right)$
 $L_{I}=L_{I}+\sum_{i}\ln p_{N}\left(\alpha_{i}|0,I\right)+\ln p_{N}\left(\beta_{i}|0,I\right)$
 # Define the “entropy part” of the ELBO function $L_{I}=L_{I}-N\cdot \ln p_{N}\left(z_{I}|h_{1,\mu }^{\prime },h_{1,\sigma }^{\prime }\right)$
 $L_{I}=L_{I}-\sum_{i}\ln p_{N}\left(\alpha_{i}|\lambda_{\alpha_{i},\mu },\lambda_{\alpha_{i},\sigma }^{2}\right)+\ln p_{N}\left(\beta_{i}|\lambda_{\beta_{i},\mu },\lambda_{\beta_{i},\sigma }^{2}\right)$
 return $L_{I}$


### 5.3. Discussion

Although the different variational techniques we have presented in this section can be used to make inference over probabilistic models with deep neural networks, each of them presents different trade-offs which should be taken into account. The black-box methods presented in Section 5.1 are extremely powerful in theory, as they make only few assumptions about the probabilistic model. However, this comes at a price: a high variance is introduced when the gradient is estimated using Monte Carlo sampling, and this can strongly hinder the convergence of the underlying optimization method. In this sense, pathwise gradients (Section 5.1.1) should be preferred to score function gradients (Section 5.1.2), because the former produces less noisy gradient estimates than the latter. Score function gradients should only be used when the pathwise gradient estimator is not applicable. Furthermore, as commented before, they have to be implemented using the variance reduction technique [24,86,92,93].

Amortized variational inference (Section 5.2) is usually the default choice. Although amortized variational inference reduces the flexibility of the variational approximation, it greatly improves the computational efficiency, to the point that most standard LVMs cannot be used for analyzing any meaningful data sample without this technique. The lack of flexibility of amortized variational inference can be alleviated by the use of more expressive mapping functions (i.e., more powerful neural networks).

## 6. Probabilistic Programming Languages

One of the main reasons for the wide adoption of deep learning has been the availability of (open-source) software tools containing robust and well-tested implementations of the main building blocks for defining and learning DNNs [34,35]. Recently, a new wave of software tools have appeared, building on top of these deep learning frameworks in order to accommodate modern probabilistic models containing DNNs [29,30,31,32,33,96,97,98]. These software tools usually fall under the umbrella term *probabilistic programming languages* (PPLs) [37,38], and support methods for learning and reasoning about complex probabilistic models. Although PPLs have been present in the field of machine learning for many years, traditional PPLs have mainly focused on defining languages for expressing (more restricted types of) probabilistic models [8] with only little focus on issues such as scalability. The advent of deep learning and the introduction of probabilistic models containing DNNs has motivated the development of a new family of PPLs offering support for flexible and complex models as well as scalable inference. Some of the main PPLs supporting the definition of models with DNNs are listed below.

- Edward2 [29,30], developed by Google, is a fast Python PPL built over TensorFlow-probability [34,78]. This framework is compatible with neural networks defined with Keras [99].
- InferPy [32,33] is a Python package built on top of Edward which focuses on the ease of use. It provides a compact and simple way to code probabilistic models with DNNs, at the expense of slightly reducing expressibility and flexibility.
- Pyro [31], developed by Uber, is based on Pytorch and allows for the definition of probabilistic models with DNNs in Python [35].
- PyMC3 [96] is a PPL written in Python that uses Theano [100] as calculus framework.
- Stan [97] is a PPL in C++ for statistical modeling and high-performance statistical computation. Even though the integration with DNNs is not natively supported, an extension for this was proposed [101].
- Turing.jl [98] is a Julia library for probabilistic programming inference. Originally, Mote Carlo methods were only considered, but recent releases of this library also provide support for variational inference.

There are other examples of PPLs, but these alternatively PPLs do typically not support DNNs in the model specifications. Examples of such languages include: Birch [102] is a C++ library with inference algorithms based on Sequential Monte Carlo (SMC); Bean Machine [103] is a declarative PPL in Python with a special focus on compositional and block inference; Infer.net [104] is a framework for running Bayesian inference in graphical models which can also be used for probabilistic programming.

## 7. Conclusions and Open Issues

In this paper, we have discussed the recent breakthroughs in approximate inference for PGMs. In particular, we have considered variational inference (VI), a scalable and versatile approach for doing approximate inference in probabilistic models. The versatility of VI enables the data analyst to build flexible models, without the constraints of limiting modeling assumptions (e.g., linear relationship between random variables). VI is supported by a sound and well-understood mathematical foundation and exhibits good theoretical properties. For instance, VI is (theoretically) guaranteed to converge to an approximate posterior *q*, contained in a set of viable approximations Q, that corresponds to a (local) maximum of the ELBO function, as defined in Equation (8). Nevertheless, variational inference often encounters difficulties when used in practice. Different random initializations of the parameter space can have significant effect on the end-result and, unless extra care is taken, issues wrt. numerical stability may also endanger the robustness of the obtained results. More research is needed to develop practical guidelines for using variational inference.

As the power of deep neural networks has entered in PGMs, the PGM community has largely responded enthusiastically, embracing the new extensions to the PGM toolbox and used them eagerly. This has lead to new and interesting tools and models, some of which are discussed in this paper. However, we also see a potential pitfall here: The trend is to move away from the modeling paradigm that the PGM community has traditionally held in so high regard and instead move towards catch-all LVMs (like the one depicted in Figure 1). These models “*let the data speak for itself*”, but at the cost of interpretability. PGMs are typically seen as fully transparent models, but risk becoming more opaque with the increased emphasis on LVMs parameterized through deep neural networks and driven by general purpose inference techniques. Initial steps have, however, already been made to leverage the PGM’s modeling power also in this context (e.g., Ref. [68] combines structured latent variable representations with non-linear likelihood functions), but a seamless and transparent integration of neural networks and PGMs still requires further developments: Firstly, in a PGM model where some variables are defined using traditional probability distributions and others use deep neural networks, parts of the model may lend itself to efficient approximative inference (e.g., using VMP as described in Section 2.4), while others do not. An inference engine that utilizes an efficient (mixed) strategy approach for approximate inference in such models would be a valuable contribution. Secondly, VI reduces the inference problem to a continuous optimization problem. However, this is insufficient if the model contains latent categorical variables. While some PPLs, like the current release (Pyro version 1.5.1.) of Pyro [31], implements automatic enumeration over discrete latent variables, alternative approaches like the Concrete distribution [105] are also gaining some popularity. Thirdly, with a combined focus on inference and modeling, we may balance the results of performing approximate inference in "exact models" and performing exact inference in "approximate models" (with the understanding that all models are approximations). Here, the modeling approach may lead to better understood approximations, and therefore give results that are more robust and better suited for decision support.

## Data Availability

The running examples of the paper together with other basic models are available at https://github.com/PGM-Lab/ProbModelsDNNs.
